# Analysis of Pacing Strategies in AMRAP, EMOM, and FOR TIME Training Models during “Cross” Modalities

**DOI:** 10.3390/sports9110144

**Published:** 2021-10-20

**Authors:** Levy Anthony de-Oliveira, Juan Ramón Heredia-Elvar, José Luis Maté-Muñoz, Juan Manuel García-Manso, José Carlos Aragão-Santos, Marzo Edir Da Silva-Grigoletto

**Affiliations:** 1 Functional Training Group, Post Graduate Program in Physical Education, Department of Physical Education, Federal University of Sergipe, São Cristóvão 49100-000, Brazil; levyanthonysouza@gmail.com; 2 Department of Physical Activity and Sports Science, Alfonso X El Sabio University, 28691 Madrid, Spain; jelvaher@uax.es; 3 Department of Radiology, Rehabilitation and Physiotherapy, Complutense University of Madrid, 28040 Madrid, Spain; jmate03@ucm.es; 4 Laboratory of Analysis and Training Planning, Physical Education Department, University of Las Palmas de Gran Canaria, 35016 Las Palmas de Gran Canaria, Spain; jgarciamanso@gmail.com; 5 Center of Biological and Health Sciences, Post Graduate Program in Health Sciences, Department of Medicine, Federal University of Sergipe, São Cristóvão 49100-000, Brazil; prof.josecarlosaragao@gmail.com

**Keywords:** strength, endurance, cross training, pacing, functional fitness, health, performance

## Abstract

Empirically, it is widely discussed in “Cross” modalities that the pacing strategy developed by an athlete or trainee has a significant impact on the endurance performance in a WOD in the AMRAP, EMOM, or FOR TIME model. We can observe at least six pacing strategies adopted during the cyclical modalities in the endurance performance in the scientific literature. However, besides these modalities, exercises of acyclical modalities of weightlifting and gymnastics are performed in the “Cross” modalities. These exercises may not allow the same pacing strategies adopted during cyclic modalities’ movements due to their motor characteristics and different intensity and level of effort imposed to perform the motor gesture. In addition to the intensity and level of effort that are generally unknown to the coach and athlete of the “Cross” modalities, another factor that can influence the adoption of a pacing strategy during a WOD in the AMRAP, EMOM, or FOR TIME model is the task endpoint knowledge, which varies according to the training model used. Thus, our objective was to evaluate situations in which these factors can influence the pacing strategies adopted in a self-regulated task with cyclic and acyclic modalities movements during an endurance workout in the AMRAP, EMOM, and FOR TIME model. Given the scarcity of studies in the scientific literature and the increasing discussion of this topic within the “Cross” modalities, this manuscript can help scientists and coaches better orient their research problems or training programs and analyze and interpret new findings more accurately.

## 1. Introduction

Pacing strategies refer to the approach adopted by an individual consciously or subconsciously to control and use energy stores, seeking to distribute work and energy during a physical effort to avoid early fatigue and achieve the best possible performance [1]. In the scientific literature, we can find at least six pacing strategies: negative, positive, all-out, even, parabolic, and variable [1]. The use of one or the other will depend on the task duration and its specific objective (win, overcome typical rivals, or respond effectively to the competition characteristics, for example). Any of them is conditioned by their metabolic dependence and by the athlete’s rating of perceived exertion. Both considerations must always be taken into account, as they are responsible for the degree of fatigue that the individual senses, conditioning their response. 

Such strategies have been extensively studied during endurance performance (performance during full-body, dynamic exercise that involves continuous effort and lasts 75 s or more [2]), mainly after the central governor model postulation. The authors tried to explain how performance regulation occurs during exercise. In this model, it is proposed that the central nervous system regulates performance through constant changes in the number of motor units recruited from active muscles in response to several conscious and subconscious factors present before and during exercise [3]. This complex regulation aims to maintain homeostasis and prevent a catastrophic physiological failure from happening, and the result of this regulation is the pacing strategy developed during physical exercise [4].

According to the central governor model, endurance performance regulation during a self-paced exercise in cyclical modalities is achieved through a combination of afferent feedback (which generates a conscious rating of perceived exertion (RPE) and an anticipatory prediction (which produces a “model” RPE to which the conscious RPE is compared) [5]. The central nervous system regulates performance to ensure that this conscious RPE does not increase excessively at any exercise stage, leading to premature termination. Importantly, to prevent conscious RPE from exceeding acceptable levels, there must be some expected or “acceptable” RPE at any stage during an exercise against which conscious RPE can be continually compared. This expected or “acceptable” RPE is the so-called RPE template generated due to previous experience with training and knowledge of the duration or distance of the exercise [5,6]. It is also worth noting that this RPE template is a theoretical construct that is difficult to measure, but it is necessary to interpret the conscious RPE [5].

The anticipatory component is influenced by previous exercise experience, knowledge of the distance or final duration of the exercise, physiological, psychological, and contextual information (increased skin temperature, levels of motivation, and presence of competitors, for example). Based on these factors, the athlete automatically selects an initial effort intensity that is considered optimal for the expected duration or distance of the exercise [5]. At the exercise beginning, the conscious RPE is compared to the RPE template (or previous experience with the task) throughout the entire exercise. This RPE template represents an RPE that the athlete finds acceptable for any stage during the exercise. According to this model, the athlete regulates the RPE, through changes in exercise performance, from changes in the degree of recruitment of the skeletal musculature to ensure that the conscious RPE is acceptable. Finally, this regulatory system will prevent harmfully or limiting physiological changes that occur before the end of the exercise while optimizing performance [5]. 

In contrast, another model was proposed, the psychobiological model, in which more importance was given to psychological and motivational factors for the endurance exercise performance regulation [7]. This model explains the inability to maintain a physical task based on psychological exercise (in)tolerance. According to the psychobiological model, the self-paced tasks during endurance performance is based on five psychological factors: (1) RPE, (2) potential motivation, (3) knowledge of the total distance/duration to perform the task, (4) knowledge of the remaining distance/duration, and (5) previous experience of perceived exertion during exercises of varying intensity and duration [8]. 

However, concerning factors 3 and 4, in the “Cross” modalities, the endurance performance is usually stimulated during the central part of the training session, commonly called workout of the day (WOD). The movements selected to the WOD are those in which high loads can be moved and can be performed at high speed and for long distances [9]. The movements of such modalities are constantly varied and can be performed at high intensity, generating a high degree of fatigue (7). We refer to the “Cross” modalities as all of the training modalities are popularly called “Cross” (such as CrossFit^®^, Functional Fitness, High Intensity Functional Training, Cross-Training, among other training modalities).

## 2. “Cross” Modalities and Pacing Strategies

### 2.1. “Cross” Modalities

In the “Cross” modalities, movements of three categories are performed: weightlifting (W), gymnastics (G), and monostructural metabolic conditioning (MMC) [9,10]. The W category contains the so-called “strength exercises” that involve any weight lifting, generally from the acyclical modalities of the Olympic W and powerlifting, such as the snatch, clean and jerk, deadlift, and others. The G category contains the so-called “strength exercises” of the acyclic modality of G and calisthenics, such as pull-ups, muscle-ups, handstands, and others. The MMC category contains the so-called “endurance exercises”, movements of cyclical modalities, such as running, rowing, and so on, in addition to rope jumping. Thus, it will often not be possible to know the (factor 3) total distance/duration of the task and the (factor 4) remaining distance/duration during the WOD due to the exercise characteristic, coming from a modality that is not cyclic (W or G) and due to the model used to execute the WOD.

The WOD is typically performed with task and time priority training models. Three models highly used in the WOD are the AMRAP (as many reps/rounds as possible), EMOM (every minute on the minute), and FT (for time). In the AMRAP model, the largest possible task volume (number of repetitions or rounds) is performed in a fixed time interval. In the EMOM model, the exercise must be performed within one minute, while in the FT model, the proposed task must be completed in the shortest time possible [9,11]. In addition to the AMRAP, the EMOM and FT models can also have a fixed total time interval or a time cap for task conclusion. 

In these three models, it is possible to know the endpoint by the total task duration (as in AMRAP and EMOM with fixed total time, for example) or by the total task volume (as in FT, for example), which during a WOD with movements of the acyclical modalities (W and G), it can be determined by the number of repetitions, sets, and rounds and with cyclical modalities movements (of the MMC) by the time of effort execution or by the distance covered, for example. In the AMRAP and EMOM without fixed total time models, it is only possible to know the total time and the partial time of one minute, respectively, but not the total volume of the task, which will depend on the athlete’s current performance capacity. In the FT model with and without a time cap, the total task volume is known. However, in the FT model with a time cap, the time limit for carrying out the task is known, as well as in the EMOM with total time fixed, in which we have the greatest knowledge of the task’s endpoint, not only by knowing the total time to perform the task, as well as the partial time of one minute and the total volume of the task (Table 1). Thus, knowledge of the endpoint dependent on the model used for the WOD can result in different pacing strategies adopted during the AMRAP, EMOM, and FT models. 

In addition to this factor, the pace used during a task is dependent on numerous factors, as mentioned elsewhere [1,12], mainly during the performance of cyclical modalities. However, with the acyclical modalities’ movements, other factors can exert extreme importance on the pace used during the task performance in the WOD. These factors influence the training load character and global magnitude [13,14], usually not known by the coaches and athletes of “Cross” modalities. They are intensity, level of effort (proximity to muscle failure), and exercise type concerning its motor characteristics (simple, combined, sequential, or complex).

Thus, we aimed to evaluate situations in which these factors (task endpoint knowledge, exercise intensity, level of effort, and exercise type) can influence the pacing strategies adopted in a self-regulated task with movements of the cyclic (from MMC) and acyclic (from W and G) modalities during an endurance workout in the AMRAP, EMOM, and FT model in the “Cross” modalities. In addition, we provide recommendations on possible pacing strategies that might be more conducive to performance in the health and competitive context. We focused on analyzing these strategies in acyclical modalities movements since many studies exist in the scientific literature about pacing strategies in cyclical modalities. In addition, the increasing discussion of this topic within “Cross” modalities can help scientists and coaches guide their research problems or training programs better and analyze and interpret new findings more accurately. 

### 2.2. Pacing Strategies in a Short, Medium, and Long Duration WOD

To avoid confusion, it is noteworthy that, in addition to pacing strategies, there is a general strategy for performing the WOD, which conditions how the practitioner intends to perform the WOD and depends on each individual’s current performance capacities. The WOD strategy is a plan that directs how the WOD should be carried out. It is often programmed and prescribed by the coach for athletes of different fitness levels. The analysis of the potential pacing strategies was performed separated by short, medium, and long duration WOD, since the time available to apply force is one of the main factors determining the degree of applied force [15] and, consequently, the WOD strategy and the pacing strategy adopted. In addition, we separated the topics by the training model (AMRAP, EMOM, and FT) for discussion, as we believe that the model used can also generate different WOD strategies and different pacing strategies due to different endpoint task knowledge. The time intervals we propose to demarcate the short, medium, and long duration WODs is our proposal based on the benchmark WODs [16,17].

It is worth emphasizing that, within each WOD, there may be more metabolically dependent movements, with significant endurance appeal, like the movements of cyclical modalities or more neuromuscular ones, like the movements of acyclical modalities. Thus, it may be that the pacing strategy varies from one movement to another and is not the same for all exercises present in a WOD. For example, the pacing strategy used for running may not be the same for performing a squat. Possibly this is due to different intensities for each exercise. Thus, we emphasize that exercise intensity can be the primary conditioning variable of pacing control (deliberate increase or decrease in movement velocity), especially during exercise in acyclic modalities (W and G), which is usually necessary to apply more force to perform the movements.

Understanding the exercise intensity, such as the degree of effort developed in the first repetition [15], the pacing control during exercises of acyclical modalities performed with heavy loads will become challenging to accomplish without the inclusion of rest intervals, for the applied force recovery. For example, even if an athlete wants to increase velocity when lifting a heavy load during a WOD, it will not do so without a recovery interval necessary for this increase. The greater the number of repetitions performed, without a rest interval, the greater the level of effort and the closer to the minimum velocity threshold the athlete will be [15]. The minimal velocity threshold is the mean concentric velocity produced on the last successful repetition of a set to failure performed at maximal intended velocity [18]. Against heavy loads or unintentionally attaining velocities close to the minimum velocity threshold, it may not be possible to control the pacing without the necessary recovery interval, thus only resulting from the individual’s current performance capacity. Thus, in each topic, we discussed the possibility of including programmed rest intervals to achieve better performance during a WOD.

It is worth noting that, during the WOD in the “Cross” modalities, we rarely see that the coach or the athlete know the relative intensity that represents the load to be moved (either just the bodyweight itself or with the addition of an external resistance). This knowledge is essential for the proper control of the training load for controlling the pacing strategy adopted during the WOD. The coach and the athlete could have this information if the movement velocity could be measured.

In addition to the intensity and level of effort, the exercise type in W and G categories can also influence the pacing strategy adopted since, for the same volume and relative intensity, not all exercise types assume the same degree of effort. The greater the number of active muscle groups and the exercise coordination requirement, we can assume that the greater the degree of a global effort that the exercise will represent [13]. For example, suppose the same volume and relative intensity are prescribed in a WOD, for a snatch (sequential or complex exercise) and for a push-up (simple exercise, in which only one body hemisphere is dynamically involved). In that case, the sequential exercise will assume a more significant real/internal load than the simple exercise. Thus, all these factors may influence the pacing strategy adopted in each exercise.

#### 2.2.1. Short-Duration WOD (≤10 min)

**Figure 1:**
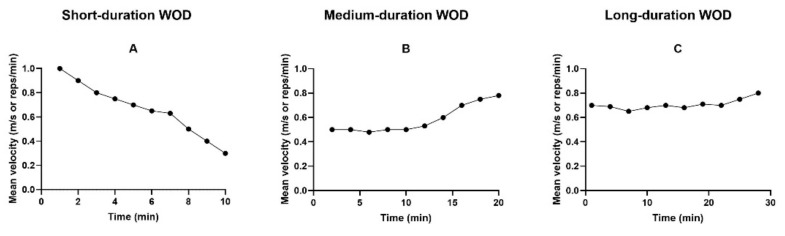
Illustration of all-out/positive (**A**), negative (**B**), and even (**C**) pacing strategies proposals applied in a short, medium, and long duration WOD.

With the short time to perform the task, the WOD strategy may be mainly with the all-out or positive pacing strategies in each exercise. These strategies occur when there is a gradual velocity loss from beginning to the task end (Figure 1A), with the difference that, in the all-out, there is the intention to perform the task as quickly as possible [1]. Velocity starts high and progressively decreases over time due to applied force loss and fatigue generated. In short events (less than 2 min) of cyclic modalities, Foster et al. [19] found that the pattern of pacing strategy adopted is the all-out strategy. Some studies have shown that all-out or positive pacing strategies result in a more significant accumulation of fatigue-related metabolites (inorganic phosphate and hydrogen ions, for example) and high RPE in the early stages of cyclic modalities [1,20,21,22]. However, when considering a short duration WOD less than or equal to 10 min and when faced with a task longer than 2–3 min, we will hardly find an all-out strategy being performed when exercising at maximum intensity. 

Although some authors consider all-out efforts above this duration in the “Cross” modalities, this does not seem correct from a terminological point of view. When performing an all-out effort, the phosphagen and glycolytic systems will predominate in the energy supply, indicating a decrease in the applied force in a short time. The all-out effort during cyclical modalities is characterized by reaching peak power in the first 5 to 10 s, followed by a progressive decline in power output until the end of the effort [23]. Thus, when performing an effort above 2–3 min, it is still possible to produce high velocity and power, but they will never be maximum.

In the movements of the acyclical modalities of W and G, such strategies can be adopted when the WOD sets are performed in a traditional configuration, that is, without intra-set rest intervals. In this scenario, the level of effort becomes increasingly higher throughout the repetitions, and the velocity drops as a function of the applied force loss, with velocity being a reliable indicator of the degree of fatigue developed during the sets [15,24]. Thus, in a competitive context, these strategies seem to be the most logical to be used in such movements if the athlete wants to achieve the best possible performance during a short-duration WOD and if the physical condition allows for performing all sets without rest. 

If we analyze scientific studies in which the responses of different set configurations on the strength exercise velocities were observed [25,26,27], we will often see the adoption of a variable pace. In cyclical modalities, the variable pace strategy is one in which we observe fluctuations in velocity throughout the exercise [1] (gradual velocity loss, followed by an increase in a constant cycle). For the variable pace to occur in strength exercises, there must be enough intra-set or inter-set recovery intervals so that an ATP resynthesis can occur that allows the velocity to increase after a gradual loss, contributing to the appearance of the variable pace. Thus, when we are not dealing with elite athletes, it seems to us that most people will perform the WOD adopting a variable pace in the acyclic modalities’ exercises, since we will see some rest interval within the WOD, to allow an ATP resynthesis, in order to complete the task. Finally, it is noteworthy that the error in selecting the appropriate pace in a short WOD will be crucial, as the individual may not have time to recover and achieve the best performance within this time interval to complete the WOD.

##### As Many Repetitions/Rounds as Possible (AMRAP)

The AMRAP model aims to achieve the highest number of repetitions or rounds during the task. In an interesting study, Iglesias-Soler et al. [28] compared the maximum number of repetitions in three traditional sets with 3 min of inter-set rest on the squat, with the inclusion of inter-repetition rests equivalent to the traditional set work–rest ratio (which led to a 45-s rest, on average). They verified that the inclusion of rests between each repetition led to a number of repetitions, on average, nine times greater than the traditional sets. Thus, the inclusion of programmed rest inter-repetitions can allow a more significant number of repetitions and better maintenance of mechanical performance in resistance exercises performed at high intensity, being a strategy to be considered by coaches for the development of muscular endurance [28].

In the AMRAP model, it is possible to know the total WOD time. With the correct selection and distribution of intra-set rests, this inclusion may lead to better performance than traditional sets with self-selected rest intervals. Such inclusion may not allow a drastic reduction of the applied force, allowing minor velocity loss, leading to a total rest time lesser than when resting when necessary. The strategy of incorporating a recovery within the set (intra-set rest), with rest intervals at each repetition (inter-repetition rest) or each set of repetitions (cluster set), is efficient to attenuate the velocity and power loss during strength exercises, especially with moderate and high loads, in which fatigue can impair neuromuscular performance [29], changing the quality of each movement [30]. However, to achieve better endurance performance during the AMRAP model, it seems to us that such a strategy will only be valid to be implemented if it allows for less total rest time (time in which the task is not performed) and higher task execution velocity (when the relative intensity that the absolute load represents is smaller).

The planned inclusion of these rest intervals goes against the model’s objective since the intention is to perform the maximum number of repetitions or rounds during the task. However, in a non-competition context, the determination of intra-set or inter-set rests programmed by the coach can be an effective strategy for maintaining mechanical performance and movement technique, leading to less velocity loss with less fatigue and without losing the gamification of the model during the WOD. Furthermore, Iglesias-Soler et al. [28] also showed that the inclusion of inter-repetition rests when lifting high loads can be an efficient strategy for muscle hypertrophy since it can generate a higher volume load. This strategy can be helpful in the skill block of the training session of “Cross” modalities, for example.

##### For Time (FT)

When it is not possible to know the total time of the task, as in the FT model, it may be that the pacing strategies are not well designed, requiring greater previous experience with the type of task. Thus, it may be that the presence of a time limit for the realization of the FT model helps the athlete to obtain a better adjustment and control of the pace during the WOD. With this knowledge, the athlete could also use the RPE to control the pace, comparing the conscious RPE with the RPE template that should be acceptable (as proposed by the central governor model during cyclical modalities) based on the time remaining to completion of the WOD.

Furthermore, Hardee et al. [31] showed that the inclusion of pauses between repetitions of 20 s or more maintains the technique in the power clean exercise even when performed at high intensity, which can lead to an attenuation of fatigue and loss of applied force, which was not found when performing traditional series. However, this inclusion goes against the model’s objective of performing the task in the shortest time possible. In the health context, this inclusion of programmed rests can be of great value when performing sequential exercises, such as the power clean, which requires high technical proficiency. As it is known that neuromuscular fatigue modifies the movement’s biodynamics [32], it may lead to injury during the session and in subsequent training sessions if the proper recovery is not met [32,33]. 

In the context of competitive performance, these programmed rests can lead to worse performance in this model, as they can generate more time to complete the task. In other words, trying to program rests leads to a greater chance of error in achieving a better performance than not programming rests, in which the athlete tries to complete the task in less time by self-regulating efforts. However, González-Hernádez et al. [27] found a set configuration that allowed a shorter total time to perform a strength training session. They compared the performance of three traditional sets of 10 maximum repetitions with 5 min inter-sets in the full squat with other set configurations that included intra-set rests, with the same task volume (30 total repetitions) and intensity. They found that the configuration with the inclusion of 15 s of recovery between each repetition in a single set of 30 repetitions led to a shorter total session time (8.75 min) when compared to all other configurations (greater than 11 min, approximately) and with lower fatigue indicators than when performing traditional sets with inter-set rest intervals. 

Thus, it seems that not getting close to failure during the sets with inter-repetition recovery intervals can be interesting to achieve good performance. Both in the competitive context (not including elite athletes) and a health context, the inclusion of inter-repetition rests may lead not only to better performance by reducing the total time of the task but also to greater adherence to the training program due to the lower metabolic and mechanical fatigue generated compared to traditional sets until failure. This inclusion can lead to lower perceptions of discomfort and displeasure during the training program. However, this inclusion will only be valid for the competitive context if it generates less time to perform the task.

For example, in a benchmark WOD like the GRACE, 30 repetitions of clean and jerks are performed on the FT model. We can find practitioners who cannot perform a single series of 30 repetitions in the health context, without intra-set rest intervals, even with the adapted load intensity. Thus, the inclusion of programmed rests by the trainer so as not to lose the gamification of the model can be an excellent strategy to maintain the technique and reduce the injury risk, especially during sequential exercises. Some interventions can be: (1) the inclusion of inter-repetition pauses (15 s, for example) that allow the performance of 30 repetitions in a single set; (2) the inclusion of inter-set pauses when performing five sets of six repetitions; or (3) rests between sets of reps in a descending pyramid configuration of 12-8-6-4 reps, for example. It is worth noting that the load intensity will condition the pause magnitude needed to maintain mechanical efficiency [34]. Thus, the rest intervals’ magnitude will depend on the athlete’s level of performance and the intensity of the exercise.

##### Every Minute on the Minute (EMOM)

As in this model, the time interval to complete the exercise or task will always be one minute, and the correct WOD and pacing strategy will be even more crucial for the WOD performance because of the error in selecting the proper pace in a short WOD where a movement or task that must be completed within one minute may result in early task completion. Most of the time, the athlete will be “forced” to rest within the minute. Thus, it is very likely that this model allows for a variable pacing strategy throughout the task. 

Considering the study results above, the inclusion of programmed rest intervals compared to not including these intervals could also lead to better performance in an EMOM. For example, in a 5-min EMOM where the task is to complete six burpees within each minute, we can know the total WOD time (5 min) and the remaining over the WOD, the time to complete the task (each one-minute interval), the total task volume (30 burpees) and the volume remaining over the WOD. In this example, depending on the athlete’s previous experience and fitness level, we can have the following pacing strategies: (1) perform the six repetitions quickly and rest for the remaining time; if the practitioner does not have much strength to complete the task quickly or previous experience with this type of task, other strategies would be (2) to include inter-repetition pauses, which can be marked in the total time, in order to do one repetition every 10 s, while resting for the time remaining in the 10-s interval to complete the task; (3) include the rests in a set of repetitions, performing cluster sets, so that the athlete performs three burpees, rests a few seconds and completes the other three, for example. 

Finally, the inclusion of a predetermined total time in this model can also help the athlete control his pace better, knowing the remaining duration for completing the WOD. With this knowledge, the athlete could also use the RPE to control the pace, comparing the conscious RPE with the RPE template that should be acceptable (as proposed by the central governor model), based on the time remaining to complete the WOD. If there is no predetermined total time to perform the EMOM and it is only possible to know the time remaining to complete one minute in each round, it seems to us that regulating the pace will be a more difficult task for the athlete and for the coach to perform an adequate redistribution of rest intervals. Thus, the more it is possible to know the task endpoint, especially in the EMOM model, the better the pace regulation and control during the task. 

#### 2.2.2. Medium-Duration WOD (>10 min and ≤20 min)

With the increase in the time available for performing the WOD, the need to control the pace to achieve the best possible performance in the AMRAP, EMOM, and FT models becomes even more evident. With a longer time interval for ending the WOD, the greater the possibility of having unprogrammed rest intervals during the WOD. Of course, some elite athletes can perform a medium duration WOD without rest intervals in some exercises. However, in most athletes in the health context, when compared to a short duration WOD, there is a greater possibility of having unprogrammed rest intervals in a medium duration WOD.

In the competitive context, the negative pacing strategy (the one in which the athlete’s velocity increases throughout the task [1] (Figure 1B)) is much commented on in professional practice to achieve better performance. It could allow less reduction in the depletion rate of energy stores and less accumulation of metabolites (inorganic phosphate and hydrogen ions, for example) during the beginning of WOD, especially during cyclical modalities. In addition, it is noteworthy that a determining factor in maintaining this neuromuscular work is the maintenance of the nerve impulse, which makes the quality of the sodium-potassium pump functioning a pivotal role to ensure the transition of the nerve impulse to the motor endplate [35,36]. Adopting a more conservative pace maintaining a moderate velocity from the beginning to the middle of the WOD, followed by an increase in velocity, moderate initially and much higher towards the task end, can be a good strategy, as it can allow the development of the end-spurt, enabling better performance. Studies [37,38,39] have shown that this phenomenon of increased movement velocity minutes before the end of a task (end-spurt) indicates that the central nervous system can nullify the inhibitory afferent feedback of fibers and access muscle reserve capacity for better performance during cyclical modalities.

However, it may be that this phenomenon does not occur in the movements of acyclical modalities, especially if traditional sets are adopted, as it can lead to a high applied force loss, not providing the proper recovery to increase velocity, as can be seen in the studies cited previously [25,26,27]. Furthermore, the deliberate decrease in velocity in the early stages of the WOD during the negative pacing strategy assumes a more extended time under tension. Thus, for a better performance to be achieved in a competitive context, maintaining a muscle reserve for the end-spurt to happen needs to generate better performance than not adopting this strategy, generating less total rest time and higher task execution velocity. In the context of health, Da Silva-Grigoletto et al. [24] recommended that the most efficient way to prevent excessive fatigue from occurring and from maintaining correct motor mechanics at a certain intensity and volume during strength exercise is to incorporate recovery within the set, with rest intervals at each repetition or when performing cluster sets.

##### As Many Repetitions/Rounds as Possible (AMRAP) and Every Minute on the Minute (EMOM)

The same considerations made in the short duration WOD subtopic fit into a medium duration WOD, with the addition of the study’s results below that the set configuration influences the RPE. Mayo et al. [40] compared a traditional 5-set configuration with a load of 10 maximum repetitions with 3 min of inter-set rest with a configuration with inter-repetition rest in which the same number of repetitions and total rest time distributed between each repetition was performed, with an average of 25 s in the bench press and 22 s in squat. The authors found that the traditional set-to-failure configuration induced greater perceived exertion than the configuration in which the rest was distributed among repetitions, even with both configurations having the same density, volume, and load intensity. This result is quite interesting because a lower RPE can make the athlete prolong the WOD, increasing the number of repetitions or rounds, making the athlete not disengage from the task early when reaching a high or maximum RPE, for example (as explained by the psychobiological model on endurance performance [7]).

Nevertheless, performing the adequate redistribution of the rest intervals in an AMRAP or EMOM model of medium duration will not be an easy task, especially when not knowing the total task volume. With the lack of this knowledge and the longer duration of the WOD, in the health context, it will be reasonably valid for the coach to program adequate intra- or inter-set rests for producing a lower degree of effort and fatigue during the task, allowing the mechanical performance to be kept relatively constant, favoring the technique of the movements, while not losing the gamification generated in these models. For example, during a benchmark WOD such as CINDY of 20 min, it will be essential to prevent the athlete from performing sets close to failure during the WOD. However, the gamification of reaching the highest task volume should not be lost.

##### For Time (FT)

The same considerations made in the short duration WOD subtopic fit into a medium duration WOD. However, in the health context, it may be that the inclusion of short programmed rests can generate lesser feelings of discomfort and displeasure that are more relevant during a medium-duration WOD, given the longer duration. This result may lead to less metabolic, mechanical, and perceived fatigue without significant performance loss, as shown by García-Ramos et al. [25]. Their study found that the inclusion of 5 s of recovery between each repetition in 3 sets of 10 repetitions in the bench press led to lower blood lactate concentrations and less velocity loss compared to performing traditional sets with the same load intensity and inter-set rest interval. The total time of the protocol session with the 5 s of recovery between each repetition was 12.75 min, while in the traditional sets, the total time was 11.5 min. This result can be significant in the health context since the inclusion of 5 s repetitions can produce lower fatigue markers, which can keep the gamification of the model to perform the task in the shortest time possible, without significant performance loss.

#### 2.2.3. Long-Duration WOD (>20 min)

The same considerations made in the previous subtopics fit into a long-duration WOD in the AMRAP, EMOM, and FT models. However, regulating the pace during a long WOD becomes even more crucial for achieving good performance. Foster et al. [19] analyzed several observational studies to find a pattern of pacing strategies adopted during various cyclical modalities. In general, the U-shaped parabolic strategy (fast start, slower middle part, followed by a velocity increase in the final part) was most described in events lasting from ~2 min to hours. During the movements of acyclical modalities, it is difficult to believe that this strategy can happen and generate better performance since the deliberate decrease in velocity, with the selection of a moderate pace, assumes a more extended time under tension. Together with the inclusion of intra-set rests, it may not lead to improved performance by leading to a possible longer task duration in the FT model, delaying the completion of the WOD and fewer rounds in the AMRAP and EMOM models, for example.

Another strategy often found during long-duration events in cyclical sports is the even strategy. During this strategy, the velocity remains relatively constant throughout the task [1] (Figure 1C). The athlete starts the WOD with a moderate velocity that allows its maintenance until the end of the WOD. This strategy can allow a lower energy expenditure without a considerable loss of strength during a long-duration WOD. When analyzing an exercise of the acyclic modality, Tufano et al. [26] found that cluster sets of 30 s every four or two repetitions during the back squat exercise performed at high volume allowed the maintenance of velocity and power throughout the exercise (that is, it allowed the adoption of an even pace). In contrast, this was not possible during the traditional sets (a variable pace was found). Thus, it may be necessary to have more previous experience for the proper pace and rest intervals selection to execute the even strategy that allows the velocity maintenance throughout the WOD.

## 3. Final Considerations

This analysis was based on reflection on the dynamics of the efforts made and the potential fatigue generated. We hope to verify and provide specific data shortly that may help confirm or disprove our analysis and recommendations. Thus, it seems that not performing the sets until or close to muscle failure can be an efficient strategy to achieve the best performance during the AMRAP, EMOM, and FT models, both in the health context and in the competitive context. For this, programming intra-set and inter-set recovery intervals may be essential. This schedule can be attractive not only for muscle strength gains but also to generate greater predisposition to future training sessions as it generates less fatigue than performing traditional sets close to or even muscle failure.

Every time we perform a non-cyclical effort, incorporating rests within sets (intra-set rest) at each repetition (inter-repetition rest) will be crucial so that there is no significant velocity and power loss, when this is the goal, always taking into account the intensity of the exercises. This inclusion could be helpful when using high loads, like in the training session skill block. However, when the loads are not as high as during the WOD, we may use another strategy, such as cluster sets of 5, 6, or 7 repetitions per set. In addition, programming rest intervals in the AMRAP, EMOM, and FT models can be helpful for beginners or athletes at the beginning of a competitive period. It can allow maintenance of movement velocity and less loss of mechanical performance with less fatigue production.

Since pacing regulation is a trainable skill [19], we suggest that exercise professionals who use these training models include teaching pace control within their training plans and programs, especially with the use of RPE scales and the level of effort, so that athletes become more aware of the proximity of muscle failure and disengagement from the task. Thus, in addition to knowing the endpoint (total duration, number of sets, repetitions, and rounds), we as coaches and researchers must also know the relative intensity that represents the load to be moved (either just the body weight itself or with the addition of an external resistance), for the proper control of the training load. However, future studies are needed for direct analysis of the WOD and ideal pacing strategies during the practice of the AMRAP, EMOM, and FT models, especially with the presence of exercise(s) of the acyclic modalities.

## Figures and Tables

**Table 1:** Example of the different endpoint knowledge of the training models.

Training Models	Task	Set/Round Duration	Total Number of Repetitions to Perform	Total Duration
AMRAP	5 pull-ups 10 push-ups 15 squats	Dependent of the athlete’s actual performance capacity	Dependent of the athlete’s performance capacity	10 min
EMOM	5 pull-ups 10 push-ups 15 squats	1 min	300	10 min
FT	1 Set/Round of 5 pull-ups 10 push-ups 15 squats	Dependent of the athlete’s actual performance capacity	30	Dependent of the athlete’s actual performance capacity

Note: AMRAP—As Many Reps/Rounds as Possible; EMOM—Every Minute on the Minute; FT—For Time.

## Data Availability

No new data were created or analyzed in this study. Data sharing is not applicable to this article.
